# Chronic Health Conditions in Aging Individuals with Intellectual Disabilities

**DOI:** 10.3390/ijerph17093126

**Published:** 2020-04-30

**Authors:** Laura García-Domínguez, Patricia Navas, Miguel Ángel Verdugo, Víctor B. Arias

**Affiliations:** Institute for Community Inclusion, University of Salamanca, 37005 Salamanca, Spain; lauragarciad@usal.es (L.G.-D.); verdugo@usal.es (M.Á.V.); vbarias@usal.es (V.B.A.)

**Keywords:** chronic health conditions, health, intellectual disability, aging

## Abstract

Life expectancy of people with intellectual disability (ID) has increased in recent decades. However, there is little evidence of whether these extra years of life are spent in good health. The aim of this study, conducted in Spain, is to obtain information about the prevalence of chronic health conditions in people with ID over the age of 44 and compare it with that of their peers without disability. Twenty health conditions were analyzed in 1040 people with ID and 12,172 people without ID through a study of their prevalence. The findings show that chronic constipation, urinary incontinence, thyroid disorders and obesity are the most prevalent chronic diseases among individuals with ID. In addition, this population group suffers these health conditions more frequently than older adults without ID. Detection and early intervention in these health conditions will improve adequate access to social health services and subsequent treatment of aging adults with ID.

## 1. Introduction

The significant increase in life expectancy is one of the major achievements of our society during the past century. By 2050, one in six people will be over the age of 65 [1] due to a number of factors that are contributing to a decrease in mortality and birth rates [2]. In developed countries, life expectancy increases by at least two years per decade [3], and the World Health Organization [4] estimates that the average number of years that a person who reaches the age of 60 is expected to live will be of around 20 years more. Life expectancy in Spain, the country where this study is carried out, is 83.19 years [5].

The reality of a greater longevity also translates to individuals with intellectual disability (ID). In the late 1970s and early 1980s [6,7,8], the scientific literature started to report an unprecedented growth in the life expectancy of individuals with ID as a result of different factors such as improvements in care provision. At the present time, the number of adults with intellectual and developmental disabilities over the age of 60 is expected to grow from around 641,860 in 2000 to 1.2 million in 2030 [9]. Moreover, people over the age of 65 in Europe already account for 45% of people with disabilities [10]. Regarding the current situation in Spain, more than 60% of the people with ID will be older than 45 in half a decade’s time [11].

In summary, people with ID can currently expect to live almost as long as their peers without disability [12,13,14,15], although the scientific literature points out certain differences in the aging process between the two groups.

Thus, people with ID (considering the broad variability within this group) can experience aging-related health conditions earlier than the general population [16,17,18,19]. In this regard, it has been pointed out that the beginning of the aging period in this population group could start at the age of 45 [20]. This premature aging can have a negative impact on their quality of life [21], and also increases their mortality rate [13,22].

Apart from experiencing premature aging, individuals with ID are more frequently affected throughout their life by certain chronic health conditions than their peers without disability, which could affect their quality of life as they age. These conditions include cardiovascular diseases [23,24], obesity [25,26], diabetes [27,28], epilepsy [29,30,31], gastrointestinal tract anomalies such as constipation [32], kidney disease [33], osteoarticular disorders [16,34], and thyroid disorders [32,35].

Although the concept of health is broad and difficult to be captured by one single measure, the presence of chronic health conditions can precipitate early health decline in aging people with ID and greater levels of frailty [36]. However, adequate prevention and identification is complex in individuals with ID due to several factors. Some of them are related to the disability itself, like the existence of communication difficulties that complicate the diagnosis [37], but there are also contextual factors that could contribute to a higher prevalence of chronic health conditions within this group. Outstanding among the latter are difficulties to access healthcare services [38], high poverty rates [39] that are related to poorer nutrition [40], residential care settings where physical inactivity might prevail [41], high rates of polypharmacy [42], or a lack of healthcare standards for people with ID in general, and for those who are aging in particular [43]. Such factors, among others, can have a negative impact not only on the incidence of health-related problems, but also on their progression and severity through the last stage of people with ID’s lifespan.

Despite the aforementioned situation, research literature comparing the prevalence of chronic health conditions among aging individuals with and without ID is scarce. Prominent in this regard are the publications from The Irish Longitudinal Study on Ageing (TILDA) and its Disability Supplement (IDS)-TILDA, a high-impact longitudinal study carried out in Ireland [44]. Most of the scientific literature, however, has focused on describing the health status of adults with ID [28,45] or comparing the health of young adults with ID with that of their peers without disability [24,29,46]. In Spain, we have data from the POMONA-Spain study [32] on the health of people with ID over the age of 18, but does not pay specific attention to the elderly population.

Thus, the present study is carried out considering the demographic changes that are taking place in our country, as well as the paucity of literature regarding the prevalence of chronic health conditions among older adults with ID. A deeper understanding of the prevalence rates of health-related problems can lead to their early prevention and detection through improvements in healthcare practices and access to health care [17]. It could also have a positive impact on the functioning and wellbeing of people with ID who are aging.

In the light of the above, the purpose of this study is to analyze the presence of chronic health conditions in elderly population with ID. The data obtained will also be compared to national-level data from adults without disabilities reported by the Spanish National Health Survey [47]. This comparison furthers our understanding of the health risks and associated chronic health problems of older individuals with ID.

## 2. Method

### 2.1. Participants

A survey aimed at gathering information on the health status of people with ID over the age of 44 was designed. These data encompass the first sample of this study, which consists of 1040 people with ID (Table 1). Men and women are equally represented in the ID population, ranging in age from 44 to 88 years old (M = 55.29; SD = 7.34). The diagnosis of the individuals assessed was intellectual disability of unknown etiology in 64.9% of the cases, 25.5% of the sample having mild ID, 42.4% moderate ID, and 32.1% severe/profound ID. Almost half (45.8%) of the sample required extensive and generalized support.

Data on the non-ID population (Table 1) were taken from the Spanish National Health Survey 2011/12 [47]. This large-scale survey is carried out in Spain around every five years with the purpose of gathering information on citizens’ health at the national and regional level, and to plan and assess healthcare procedures. It provides information on 21,007 adults, among whom 12,172 are individuals without disability over the age of 44. 

Data corresponding to the ID population were obtained from assessments carried out by 362 informants. They were mostly reference professionals for people with ID and/or their relatives, belonging to 83 disability support organizations in 34 of the 50 Spanish provinces. Their main characteristics are detailed in Table 2.

### 2.2. Instrument

Data on the health of people with ID over the age of 44 were collected by means of a survey that was designed using information extracted from three types of document: (1) scientific literature in the area of ID, health and aging published between 2000 and 2016, which was accessed through specialized databases such as PsycINFO and PubMed; (2) already existing assessment tools on health in the general and in the ID population, such as the National Core Indicators Survey [48], the SF-36 Health Survey [49], the HoNOS scales [50] and the scale used by the research team of the Carlos III Health Institute within the framework of the POMONA-Spain project [51]; and (3) the National Health Survey of the National Statistics Institute 2011/2012 [47].

These three sources were used as a basis to draft a first version of the survey consisting of a total of 55 questions. The survey was then sent to 13 experts in the area of intellectual and developmental disabilities so that they could submit their observations and comments on the questions included. After this first round, a further 20 items that were considered relevant by the experts were added, covering aspects related to mental health and difficulties in accessing healthcare services. In a second round, the survey, consisting now of 75 items, was again assessed by the team of experts. Three questions related to women’s health during pregnancy that were regarded as too specific were removed, and the items were grouped according to the structure of the National Health Survey: (a) sociodemographic data; (b) health status of the person with ID over the age of 44 (i.e., questions related to chronic diseases, mental health and behavioral problems); (c) number of accidents; (d) health-related quality of life as measured by the EuroQol five-dimensional, EQ- [52]; (e) participation restrictions; (f) limitations in daily life activities; (g) hearing and sight characteristics; (h) use of healthcare services; (i) hospitalizations, use of emergency services and healthcare insurance; (j) medication intake; (k) preventive practices; and (l) other determinants of health (e.g., tobacco and alcohol use).

The final instrument was composed of 72 questions. Examples of items within each section are presented in Table 3.

This study was focused on the analysis of chronic health conditions diagnosed by a healthcare professional that could affect people with ID during their aging process.

### 2.3. Procedure

Initially, all the organizations (N = 1068) that are part of the four main service providers for people with ID in Spain were approached. A total of 227 (21%) showed an interest in participating in the study. Finally, 83 organizations that provide support for aging people with ID in 34 different Spanish provinces took part in the study. The survey described in the previous section was sent to the organizations either via regular post or via email, along with two other documents: an informed consent form to be returned by the professionals or relatives before their participation in the study, and an information letter stating the research objectives, method, funding sources and other relevant information concerning the study, as well as contact information where requests for further information should be sent.

This study was approved by the Bioethics Committee of the University of Salamanca, and all procedures performed in this research were in accordance with the 1964 Helsinki Declaration and its later amendments or comparable ethical standards.

Data on the non-ID population were extracted from the website of the Ministry of Health, Social Services and Equality of Spain, which gathers the microdata and syntax of the National Health Survey of the National Statistics Institute 2011/2012 [47]. The cases of people under the age of 45 were excluded.

### 2.4. Data Analysis

First, data regarding the prevalence of chronic health conditions diagnosed by a healthcare professional were obtained (Table 3).

Chi-square tests were used to estimate the statistical significance of the relationship between the likelihood of developing a chronic disease and the presence of ID, alongside Cramer’s V to measure effect size: V > 0.25 being very strong effect, V > 0.15 strong effect, V > 0.10 moderate effect, V > 0.05 weak effect, and V < 0.05 no effect [53].

Due to the difference in size between the ID sample (N = 1040) and the non-ID sample (N = 12,172), the latter was divided into 10 random subsamples so that the sample size of both groups would be similar when making comparisons, and to ensure the replicability and power of the analysis conducted. Since the contrasts performed for the 10 subsamples yielded similar results, this study only reports those of the analysis based on the total data. Finally, the analysis was again carried out between the two subsamples matched on age and sex.

Data were analyzed using IBM SPSS Statistics version 25. The significance level set by the researchers to conduct the statistical analyses was α = 0.05.

## 3. Results

Table 4 gathers the frequency of occurrence of the health conditions analyzed in people with and without ID over the age of 44. The results of the analysis on the matching samples are shown in parentheses. The most frequent chronic diseases among the aging population with ID were obesity (25.3%; *n* = 263), urinary incontinence (18.7%; *n* = 192), osteoarticular problems (16.9%; *n* = 168), hypercholesterolemia (16.6%; *n* = 166), hypertension (16.6%; *n* = 166), chronic constipation (16.2%; *n* = 165) and thyroid disorders (12.4%; *n* = 122).

When comparing the prevalence of chronic health conditions between groups, we observe that the ID population was more likely to experience urinary incontinence (18.7% vs. 6.4%; χ^2^ = 205.81; *p* < 0.001; OR = 3.34) and chronic constipation (16.2% vs. 6.5%; χ^2^ = 128.69; *p* < 0.001; OR = 2.77), the strength of the association between the study variables being moderate in both cases.

Although individuals with ID experienced a higher prevalence than the general population of thyroid disorders (χ^2^ = 25.84; *p* < 0.001; OR = 1.69) and obesity (χ^2^ = 17.32; *p* < 0.001; OR = 1.37), the effect size of the association was weak in both cases (V = 0.05 y V = 0.04).

As for the remaining health conditions, there was a higher prevalence among the general population, which was especially significant and reported moderate effect sizes in the case of osteoarticular disorders (16.9% vs. 36.9%; χ^2^ = 161.47; *p* < 001; V = 0.11); hypertension (16.6% vs. 38.4%; χ^2^ = 185.2; *p* < 0.001; V = 0.12) and chronic back pain (10.4% vs. 35.3%; χ^2^ = 258.20; *p* < 001; V = 0.14).

When carrying out the analysis with the matching samples, different results were found for some chronic health conditions. Small differences observed in the beginning, suggesting a higher prevalence among individuals without ID, were no longer significant. This was the case for cataracts (9.9% vs. 9%; χ^2^ = 0.09; *p* = 0.342; V = 0), diabetes (8.9% vs. 10.4%; χ^2^ = 2.15; *p* = 0.142; V = 0.01), and other heart diseases (7.2% vs. 7.1%; χ^2^ = 0.03; *p* = 0.84; V = 0). In urinary incontinence, the initially moderate difference between groups became stronger (18.7% vs. 3.8%; χ^2^ = 396; *p* < 0.001; V = 0.20; OR = 5.77), indicating a higher prevalence within the ID group.

In the remaining conditions, no substantial changes were observed, although differences tended to be slightly less pronounced.

## 4. Discussion

The first goal of this study was to analyze the prevalence of chronic health conditions in people with ID over the age of 44. According to the results, the most prevalent diseases among the ID population are obesity, urinary incontinence, osteoarticular disorders, hypercholesterolemia, hypertension, chronic constipation and thyroid disorders. The remaining chronic health conditions studied yielded prevalence results below 10%.

The second part of the study compared the health of older adults with ID with that of their peers without disability. The scientific literature includes several references to the higher prevalence of obesity in the adult population with ID as compared to the general population [25,28,31,32,54,55]. These data seem to be replicated in elderly individuals with ID, with their probability of presenting obesity being 1.33 times higher than in the general population (25.3% vs. 20.3%). Other chronic conditions that are mentioned in the literature as being more frequent in people with ID than in those without ID are chronic constipation [32,55,56] and urinary incontinence [32]. The data of this study reflect that the probability of suffering from chronic constipation and urinary incontinence is also higher in aging individuals with ID, with the odds being 3.67 and 5.77 times greater, respectively. Other conditions, such as thyroid disorders, also occur more often in individuals with ID who are aging (12.4% vs. 7.7%), which is consistent with the findings of other studies [24,29,32,33,46].

Other diseases (e.g., allergy, asthma, chronic back pain, diabetes, hypercholesterolemia, hypertension, lung diseases, malignant tumors, migraines, myocardial infarction, osteoarticular disorders, and stomach ulcer) report lower rates of prevalence in older adults with ID. This result is also observed in other studies that analyze the risks for hypercholesterolemia [32,45,57], lung diseases [29,35,58,59] or malignant tumors [33,45,59] in this population group.

Our data, unlike those reported by other studies [24,28,31,44,60,61,62,63,64], suggest that older adults with ID are at a lower risk of suffering from diabetes or hypercholesterolemia than the general population. After controlling for age and sex, differences between the ID and non-ID population regarding diabetes disappear. Nevertheless, further research into variables that might mediate in the development of other conditions such as hypercholesterolemia is required, since other studies support the idea that the prevalence rate is similar in both population groups [28,29,31,44,57,65].

This work is the first study conducted in Spain where the health of older adults with and without ID is compared. The higher prevalence of certain chronic health conditions among older adults with ID could contribute to explaining the increased morbidity and early mortality reported within this group [13]. These health conditions may be potentially preventable with quality medical care [33]. However, research has indicated that individuals with ID are three times more likely than the general population to die from causes that could have been easily avoided [66]. Furthermore, the scientific literature has reported that older adults with ID encounter different barriers that hinder access to appropriate medical care, among which the absence of healthcare protocols focused on their aging process stands out [38,67,68]. There is therefore an urgent need to develop preventive healthcare plans to improve the health-related quality of life of older persons with ID. These healthcare plans should be accompanied by a better understanding of the patterns of medical care use and non-use among people with and without ID [47] so possible difficulties using healthcare services can be foreseen and prevented.

This study has certain limitations that should be considered. One of them is related to the contrast statistic used, chi-square, a comparison index that is sensitive to sample size, in the same way as statistical power.

A further limitation concerns the differences observed between the two sample groups. All the individuals that made up the sample without ID lived in their own home, while a large part of the ID sample lived in residential facilities. As is stated by different authors, living in residential settings may have a strong impact on people with ID’s health [69] and quality of life [70]. Also, and given the difficulties associated with self-report for people with intellectual disability [71], the survey was completed by professionals or family members who had known the person with intellectual disability for at least 12 months. This could be a limitation to the study as the knowledge of each group on the health status of the person with ID they are evaluating may differ. Furthermore, individuals without ID completed the National Health Survey by themselves, so possible bias related to different sources of information should be also taken into account.

## 5. Conclusions

This study has assessed the health status of 1040 aging individuals with ID and has compared it with that of 12,172 older adults without ID living in Spain. This pioneering study in our country suggests that the main health problems of people with ID are chronic constipation, obesity, thyroid disorders, and urinary incontinence. These conditions are more frequently observed in this population group than in older adults without disabilities. Early detection and intervention in the case of these health conditions should be a priority for public health organizations and administrations, ensuring that elderly people with ID have adequate access to appropriate healthcare services and subsequent treatment.

This research seeks to generate scientific knowledge that can help healthcare professionals to approach the services they provide for this population group more efficiently. The ultimate goal is to succeed in ensuring that the increased life expectancy of people with ID may be accompanied by the best state of health possible and that they can therefore spend their last years with better quality of life.

## Figures and Tables

**Table 1:** Sociodemographic information of the participants in the study.

Sociodemographic Data	ID (*n* = 1040) % (*n*)	Non-ID (*n* = 12,172) % (*n*)
Age		
44–64	87.2 (907)	54.8 (6669)
65 and over	12.8 (133)	45.2 (5503)
Gender		
Male	49.5 (515)	43.3 (5265)
Female	50.5 (525)	56.7 (6907)
Place of residence		
Residential settings	53.5 (554)	-
Family home	37.7 (15)	100 (12,172)
Supported living	8.2 (85)	-
Other places of residence	0.6 (6)	-

**Table 2:** Sociodemographic data of informants.

Sociodemographic Data	% (*n*)
*Gender*	
Male	24.3 (88)
Female	75.7 (274)
*Relationship with the person evaluated*	
Professionals	58.3 (211)
Mean age	40.7
Professional position	
Management or technical positions	35.9
Caregivers	21.4
Psychologists	19.4
Educators	13.1
Social workers	6.8
Occupational therapists	1.9
Doctors	1.5
Relatives	40.0 (145)
Mean age	64.7
Family relationship	
Siblings	48.3
Parents	41.4
Other relatives	10.3
Other relationships	1.7 (6)
*Frequency of contact with the person evaluated*	
Daily	74.3
Weekly	22.0
Several times per month	3.7

**Table 3:** Examples of questions included in the survey.

Survey Section	Examples of Questions
Health status of the person with ID over the age of 44	Does the individual present any of the following chronic health diseases as diagnosed by a healthcare professional? Hypertension Myocardial infarction Other heart diseases Osteoarticular diseases Chronic back pain Allergy Asthma Lung diseases Diabetes Stomach ulcer Urinary incontinence Hypercholesterolemia Cataract Chronic constipation Cerebrovascular accidents or CVAs Migraines Malignant tumors Osteoporosis Thyroid disorders Obesity
Number of accidents	During the last twelve months, has the person with intellectual disability suffered an accident of any kind, including poisoning or burns?
Participation restrictions	During the past two weeks, has the person had to reduce or limit his or her usual activities because of any pain or symptoms?
Limitations in daily life activities	Thinking about the last six months, to what extent has the person been limited in carrying out the activities that people usually do because of a healthcare problem?
Hearing and sight characteristics	Does he or she need glasses or contact lenses? Does he or she need a hearing aid?
Use of healthcare services	How long has it been since the person have his or her last health check?
Hospitalizations, use of emergency services and healthcare insurance	In the last twelve months, has the person used an emergency service for any problem or illness? In the last twelve months, have the person not received medical assistance the he or she needed?
Medication intake	Please list all medications the person is taking, indicating which one has been prescribed by a physician
Preventive practices	Has the person ever visited a gynecologist or urologist?
Other determinants of health	Does the person smoke?

**Table 4:** Prevalence of chronic health conditions in people over 44 years with intellectual disability (ID) and without ID.

Chronic Health Condition	Power (1-β)	Intellectual Disability (%)	General Population (%)	χ^2^	*p*-Value	V Cramer	Odds Ratio (ID/No ID)
Allergy	1.00	5.7	11.3 (11.7)	29.6 (33.1)	<0.001 (<0.001)	0.05 (0.05)	0.47 (0.45)
Asthma	1.00	1.9	4.9 (4.5)	17.6 (14.8)	<0.001 (<0.001)	0.04 (0.04)	0.38 (0.42)
Cataract	1.00	9.9	17.3 (9)	36.34 (0.09)	<0.001 (**0.342**)	0.05 (0)	0.52 (1.1)
Chronic back pain	0.94	10.4	35.3 (33.4)	258.2 (223)	<0.001 (<0.001)	0.14 (0.15)	0.21 (0.23)
Chronic constipation	0.97	16.2	6.5 (5)	128.69 (196)	<0.001 (<0.001)	0.1 (0.14)	2.77 (3.67)
CVAs	1.00	1.6	1.9 (1.3)	0.2 (0.5)	**0.655 (0.483)**	0.01 (0)	0.86 (1.21)
Diabetes	1.00	8.9	13.4 (10.4)	17.2 (2.15)	<0.001 (**0.142**)	0.04 (0.01)	0.62 (0.85)
Hypercholesterolemia	1.00	16.6	31.2 (29.1)	94.2 (70.5)	<0.001 (<0.001)	0.09 (0.08)	0.43 (0.48)
Hypertension	1.00	16.6	38.4 (31.7)	185.2 (95.6)	<0.001 (<0.001)	0.12 (0.10)	0.32 (0.42)
Lung disease	1.00	3.9	7.2 (5.8)	15.44 (6.4)	<0.001 (0.011)	0.04 (0.02)	0.52 (0.65)
Malignant tumors	0.98	2.4	5.2 (4.3)	14.4 (8.1)	<0.001 (0.004)	0.03 (0.02)	0.45 (0.54)
Myocardial infarction	1.00	0.6	3.4 (2.4)	22.81 (13.4)	<0.001 (<0.001)	0.04 (0.03)	0.38 (0.25)
Migraines	0.90	5.3	11 (11.1)	31.09 (31.5)	<0.001 (<0.001)	0.05 (0.05)	0.45 (0.44)
Obesity	1.00	25.3	19.8 (20.3)	17.3(13.8)	<0.001 (<0.001)	0.04 (0.037)	1.37 (1.33)
Osteoarticular disorders	0.86	16.9	36.9 (28.7)	161.47 (63.3)	<0.001 (<0.001)	0.11 (0.08)	0.34 (0.50)
Osteoporosis	1.00	6.8	8.6 (6.5)	3.44 (0.08)	**0.064 (0.76)**	0.02 (0)	0.77 (1.00)
Other heart diseases	0.57	7.2	10.7 (7.1)	11.78 (0.03)	<0.001 (0.84)	0.03 (0)	0.65 (1.03)
Stomach ulcer	1.00	2.3	6.9 (6.5)	32.2 (28.2)	<0.001 (<0.001)	0.05 (0.05)	0.31 (0.33)
Thyroid disorders	0.77	12.4	7.7 (7.7)	25.84 (26)	<0.001 (<0.001)	0.05 (0.05)	1.69 (1.70)
Urinary incontinence	1.00	18.7	6.4 (3.8)	205.81 (396)	<0.001 (<0.001)	0.13 (0.20)	3.34 (5.77)

Note: The results of the analysis on the matching samples are shown in parentheses. Non-significant relationships are highlighted in bold.

## References

[1] United Nations (2019). World Population Prospects 2019, Highlights.

[2] Yasobant S. (2018). Comprehensive public health action for our aging world: The quintessence of public health policy. J. Int. Med. Res..

[3] Mathers C.D., Stevens G.A., Boerma T., White R.A., Tobias M.I. (2015). Causes of international increases in older age life expectancy. Lancet.

[4] World Health Organization World Report on Ageing and Health.

[5] Instituto Nacional de Estadística Indicadores de Mortalidad Resultados Nacionales.

[6] Balakrishnan T.R., Wolf L.C. (1976). Life expectancy of mentally retarded persons in Canadian institutions. Am. J. Ment. Defic..

[7] Carter G., Jancar J. (1983). Mortality in the mentally handicapped: A 50 year survey at the Stoke Park group of hospitals (1930–1980). J. Ment. Defic. Res..

[8] Fryers T. (1986). Current Topic Survival in Down’s Syndrome. J. Intellect. Disabil. Res..

[9] Heller T. (2010). People with Intellectual and Developmental Disabilities Growing Old: An Overview. Impact Feature Issue Aging People Intellect. Dev. Disabil..

[10] Grammenos S. European Comparative Data on Europe 2020 & People with Disabilities. https://digitalcommons.ilr.cornell.edu/cgi/viewcontent.cgi?article=1569&context=gladnetcollect.

[11] Baroja H. (2014). Envejecimiento y Discapacidad Intelectual.

[12] Bowers B., Webber R., Bigby C. (2014). Health issues of older people with intellectual disability in group homes. J. Intellect. Dev. Disabil..

[13] Hahn J.E., Fox S., Janicki M.P. (2015). Aging among Older Adults with Intellectual and Developmental Disabilities: Setting National Goals to Address Transitions in Health, Retirement, and Late-Life. Inclusion.

[14] Lin J.-D., Lin L.-P., Hsu S.-W. (2016). Aging People with Intellectual Disabilities: Current Challenges and Effective Interventions. Rev. J. Autism Dev. Disord..

[15] Dolan E., Lane J., Hillis G., Delanty N. (2019). Changing Trends in Life Expectancy in Intellectual Disability over Time. Ir. Med. J..

[16] Haveman M., Heller T., Lee L., Maaskant M., Shooshtari S., Strydom A. (2010). Major health risks in aging persons with intellectual disabilities: An overview of recent studies. J. Policy Pract. Intellect. Disabil..

[17] Heller T., Sorensen A. (2013). Promoting healthy aging in adults with developmental disabilities. Dev. Disabil. Res. Rev..

[18] Sandberg M., Ahlstrom G., Axmon A., Kristensson J. (2016). Somatic healthcare utilisation patterns among older people with intellectual disability: An 11-year register study. BMC Health Serv. Res..

[19] Wark S., Hussain R., Edwards H. (2016). The main signs of ageing in people with intellectual disability. Aust. J. Rural Health.

[20] Barrio del Campo J.A., Árias M., Ruiz Fernández M.I., Vicente Castro F. (2007). Envejecimiento y Discapacidad Intelectual; La nueva etapa. Int. J. Dev. Educ. Psychol..

[21] Reppermund S., Trollor J.N. (2016). Successful ageing for people with an intellectual disability. Curr. Opin. Psychiatry.

[22] Hosking F.J., Carey I.M., Shah S.M., Harris T., DeWilde S., Beighton C., Cook D.G. (2016). Mortality Among Adults With Intellectual Disability in England: Comparisons With the General Population. Am. J. Public Health.

[23] Erickson S.R., Spoutz P., Dorsch M., Bleske B. (2016). Cardiovascular risk and treatment for adults with intellectual or developmental disabilities. Int. J. Cardiol..

[24] Morin D., Mérineau-Côté J., Ouellette-Kuntz H., Tassé M.J., Kerr M. (2012). A comparison of the prevalence of chronic disease among people with and without intellectual disability. Am. J. Intellect. Dev. Disabil..

[25] Hsieh K., Rimmer J.H., Heller T. (2014). Obesity and associated factors in adults with intellectual disability. J. Intellect. Disabil. Res..

[26] Salehi S., Nassadj G., Shakhi K., Hossein M., Javadipour S. (2017). The prevalence of overweight and obesity among adults with intellectual and developmental disabilities in Ahvaz, Iran. Biosci. Biotechnol. Res. Commun..

[27] Axmon A., Ahlström G., Höglund P. (2017). Prevalence and treatment of diabetes mellitus and hypertension among older adults with intellectual disability in comparison with the general population. BMC Geriatr..

[28] Emerson E., Hatton C., Baines S., Robertson J. (2016). The physical health of British adults with intellectual disability: Cross sectional study. Int. J. Equity Health.

[29] Cooper S.-A., Hughes-McCormack L., Greenlaw N., McConnachie A., Allan L., Baltzer M., McArthur L., Henderson A., Melville C., McSkimming P. (2018). Management and prevalence of long-term conditions in primary health care for adults with intellectual disabilities compared with the general population: A population-based cohort study. J. Appl. Res. Intellect. Disabil..

[30] Robertson J., Hatton C., Emerson E., Baines S. (2015). Prevalence of epilepsy among people with intellectual disabilities: A systematic review. Seizure.

[31] Wong C.W. (2011). Adults with Intellectual Disabilities Living in Hong Kong’s Residential Care Facilities: A Descriptive Analysis of Health and Disease Patterns by Sex, Age, and Presence of Down Syndrome. J. Policy Pract. Intellect. Disabil..

[32] Folch A., Salvador-Carulla L., Vicens P., Cortés M.J., Irazábal M., Muñoz S., Rovira L., Orejuela C., González J.A., Martínez-Leal R. (2019). Health indicators in intellectual developmental disorders: The key findings of the POMONA-ESP project. J. Appl. Res. Intellect. Disabil..

[33] Carey I.M., Shah S.M., Hosking F.J., DeWilde S., Harris T., Beighton C., Cook D.G. (2016). Health characteristics and consultation patterns of people with intellectual disability: A cross-sectional database study in English general practice. Br. J. Gen. Pract..

[34] Sinai A., Bohnen I., Strydom A. (2012). Older adults with intellectual disability. Curr. Opin. Psychiatry.

[35] Cooper S.-A., McLean G., Guthrie B., McConnachie A., Mercer S., Sullivan F., Morrison J. (2015). Multiple physical and mental health comorbidity in adults with intellectual disabilities: Population-based cross-sectional analysis. BMC Fam. Pract..

[36] Evenhuis H., Schoufour J., Echteld M. (2013). Frailty and intellectual disability: A different operationalization?. Dev. Disabil. Res. Rev..

[37] Domínguez J., Navas P. (2018). Deterioro cognitivo y trastorno neurodegenerativo en personas con discapacidad intelectual. Siglo Cero.

[38] Navas P., Llorente S., García L., Tassé M.J., Havercamp S.M. (2019). Improving healthcare access for older adults with intellectual disability: What are the needs?. J. Appl. Res. Intellect. Disabil..

[39] Emerson E., Hatton C. (2014). Health Inequalities and People with Intellectual Disabilities.

[40] Robertson J., Emerson E., Baines S., Hatton C. (2014). Obesity and health behaviours of British adults with self-reported intellectual impairments: Cross sectional survey. BMC Public Health.

[41] Schepens Niemiec S.L., Blanchard J., Vigen C.L.P., Martínez J., Guzmán L., Concha A., Fluke M., Carlson M. (2018). Evaluation of *¡Vivir Mi Vida!* to improve health and wellness of rural-dwelling, late middle-aged Latino adults: Results of a feasibility and pilot study of a lifestyle intervention. Prim. Health Care Res. Dev..

[42] Robertson J., Emerson E., Gregory N., Hatton C., Kessissoglou S., Hallam A. (2000). Receipt of psychotropic medication by people with intellectual disability in residential settings. J. Intellect. Disabil. Res..

[43] Bigby C. (2004). Ageing with a Lifelong Disability: A Guide to Practice, Program, and Policy Issues for Human Services Professionals.

[44] McCarron M., Cleary E., McCallion P. (2017). Health and Health-Care Utilization of the Older Population of Ireland: Comparing the Intellectual Disability Population and the General Population. Res. Aging.

[45] Cocks E., Thomson A., Thoresen S., Parsons R., Rosenwax L. (2016). Health status and use of medications by adults with intellectual disability in Western Australia. J. Intellect. Dev. Disabil..

[46] Hughes-McCormack L.A., Rydzewska E., Henderson A., MacIntyre C., Rintoul J., Cooper S.-A. (2018). Prevalence and general health status of people with intellectual disabilities in Scotland: A total population study. J. Epidemiol. Community Health.

[47] Instituto Nacional de Estadística Encuesta Nacional de Salud 2011–2012.

[48] Human Services Research Institute (HSRI), The National Association of State Directors of Developmental Disabilities Services (NASDDDS) National Core Indicators Survey.

[49] López-García E., Banegas J.R., Pérez-Regadera A.G., Gutiérrez-Fisac J.L., Alonso J., Rodríguez-Artalejo F. (2003). Valores de referencia de la versión española del Cuestionario de Salud SF-36 en población adulta de más de 60 años. Med. Clín..

[50] Ausín Benito B., Muñoz López M., Quiroga Estévez M.A. (2007). Adaptación española de las escalas de resultados para personas mayores HoNOS65+ (Health of the Nation Outcome Scales for Older Adults). Rev. Esp. Geriatr. Gerontol..

[51] Perry J., Allen D.G., Pimm C., Meek A., Lowe K., Groves S., Cohen D., Felce D. (2013). Adults with intellectual disabilities and challenging behaviour: The costs and outcomes of in- and out-of-area placements. J. Intellect. Disabil. Res..

[52] Herdman M., Gudex C., Lloyd A., Janssen M.F., Kind P., Parkin D., Bonsel G., Badia X. (2011). Development and preliminary testing of the new five-level version of EQ-5D (EQ-5D-5L). Qual. Life Res..

[53] Akoglu H. (2018). User’s guide to correlation coefficients. Turk. J. Emerg. Med..

[54] Havercamp S.M., Scott H.M. (2015). National health surveillance of adults with disabilities, adults with intellectual and developmental disabilities, and adults with no disabilities. Disabil. Health J..

[55] Martinez-Leal R., Salvador-Carulla L., Ruiz M., Nadal M., Novell-Alsina R., Martorell A., Gonzalez-Gordon R.G., Reyes M., Angel S., Milagrosa-Tejonero L. (2011). Health among persons with intellectual disability in Spain: The European POMONA-II study. Rev. Neurol..

[56] Hermans H., Evenhuis H.M. (2014). Multimorbidity in older adults with intellectual disabilities. Res. Dev. Disabil..

[57] de Winter C.F., Bastiaanse L.P., Hilgenkamp T.I.M., Evenhuis H.M., Echteld M.A. (2012). Cardiovascular risk factors (diabetes, hypertension, hypercholesterolemia and metabolic syndrome) in older people with intellectual disability: Results of the HA-ID study. Res. Dev. Disabil..

[58] Axmon A., Sandberg M., Ahlström G. (2017). Gender differences in psychiatric diagnoses in older people with intellectual disability: A register study. BMC Psychiatry.

[59] Janicki M.P., Davidson P.W., Henderson C.M., McCallion P., Taets J.D., Force L.T., Sulkes S.B., Frangenberg E., Ladrigan P.M. (2002). Health characteristics and health services utilization in older adults with intellectual disability living in community residences. J. Intellect. Disabil. Res..

[60] de Winter C.F., Magilsen K.W., van Alfen J.C., Penning C., Evenhuis H.M. (2009). Prevalence of cardiovascular risk factors in older people with intellectual disability. Am. J. Intellect. Dev. Disabil..

[61] Lin J.-D., Lin L.-P., Lee J.-T., Liou S.-W., Chen Y.-J., Hsu S.-W., Liu C.-T., Leu Y.-R., Wu C.-L. (2014). Prevalence and risk factors associated with fasting blood glucose levels in adults aged 30 years and older with disabilities: The results from an annual health check-up. Int. J. Dev. Disabil..

[62] MacRae S., Brown M., Karatzias T., Taggart L., Truesdale-Kennedy M., Walley R., Sierka A., Northway R., Carey M., Davies M. (2015). Diabetes in people with intellectual disabilities: A systematic review of the literature. Res. Dev. Disabil..

[63] McVilly K., McGillivray J., Curtis A., Lehmann J., Morrish L., Speight J. (2014). Diabetes in people with an intellectual disability: A systematic review of prevalence, incidence and impact. Diabet. Med..

[64] Wee L.E., Koh G.C., Auyong L.S., Cheong A., Myo T.T., Lin J., Lim E., Tan S., Sundaramurthy S., Koh C.W. (2014). Screening for cardiovascular disease risk factors at baseline and post intervention among adults with intellectual disabilities in an urbanised Asian society. J. Intellect. Disabil. Res..

[65] van de Louw J., Vorstenbosch R., Vinck L., Penning C., Evenhuis H. (2009). Prevalence of hypertension in adults with intellectual disability in the Netherlands. J. Intellect. Disabil. Res..

[66] Jaques H. (2014). Premature death in people with learning disabilities. Nurs. Times.

[67] Ali A., Scior K., Ratti V., Strydom A., King M., Hassiotis A. (2013). Discrimination and Other Barriers to Accessing Health Care: Perspectives of Patients with Mild and Moderate Intellectual Disability and Their Carers. PLoS ONE.

[68] Schepens H.R.M.M., Van Puyenbroeck J., Maes B. (2019). How to improve the quality of life of elderly people with intellectual disability: A systematic literature review of support strategies. J. Appl. Res. Intellect. Disabil..

[69] Martínez-Leal R., Salvador-Carulla L., Linehan C., Walsh P., Weber G., Van Hove G., Määttä T., Azema B., Haveman M., Buono S. (2011). The impact of living arrangements and deinstitutionalisation in the health status of persons with intellectual disability in Europe: A study using a health survey in 14 EC countries. J. Intellect. Disabil. Res..

[70] Sáenz I. (2018). Influencia del tipo de vivienda en la calidad de vida de las personas mayores con discapacidad intelectual o del desarrollo. Siglo Cero.

[71] Lunsky Y., Emery C.F., Benson B.A. (2002). Staff and self-reports of health behaviours, somatic complaints, and medications among adults with mild intellectual disability. J. Intellect. Dev. Disabil..

